# Risk Factors for Post-appendectomy Surgical Site Infection in Laparoscopy and Laparotomy - Retrospective Cohort Study

**DOI:** 10.7759/cureus.44237

**Published:** 2023-08-28

**Authors:** Amer Fayraq, Saif A Alzahrani, Ahmed G Alsayaf Alghamdi, Saleh M Alzhrani, Abdullmajeed A Alghamdi, Hashem B Abood

**Affiliations:** 1 Preventive Medicine, King Abdullah International Medical Research Centre, Jeddah, SAU; 2 Preventive Medicine, King Abdulaziz Medical City, Jeddah, SAU; 3 Preventive Medicine, Ministry of National Guard Health Affairs, Jeddah, SAU; 4 General Surgery, King Fahad General Hospital, Al Baha, SAU; 5 General and Colorectal Surgery, King Fahad General Hospital, Al Baha, SAU; 6 Preventive Medicine, Ministry of Health, Jeddah, SAU; 7 Medical Directorate, Saudi Royal Land Forces, Riyadh, SAU; 8 Surgery, King Fahad General Hospital, Al Baha, SAU

**Keywords:** surgical site infection (ssi), risk factors, laparotomy, laparoscopy, appendectomy

## Abstract

Background

Appendicitis is a frequent emergency condition. Surgical site infections (SSI) are a common complication of appendectomy. Despite improvements in infection control, SSIs continue to cause harm, prolonged hospital stays, and even death.

Objective

The objective of this study is to compare the risk of developing surgical site infections (SSIs) between open laparotomy and laparoscopic appendectomies in Al-Baha, Saudi Arabia.

Methods

This retrospective cohort study compared laparotomy and laparoscopy for post-operative surgical site infection among patients who underwent an appendectomy at King Fahad Hospital (KFH) in Albaha, Saudi Arabia. Medical record numbers (MRNs) of patients who met the inclusion criteria were collected to build the sampling frame. From the final sampling frame, simple random sampling using a random number generator was used to draw a representative sample. Data were collected from the surgical health records of the patients. The collected data included patients' demographics, comorbidities, presenting symptoms, ordered imaging studies, pre-operative shaving, type and duration of surgery, intraoperative findings, and signs of wound inflammation.

Results

The total number of patients included in the analysis was 256, who underwent surgery for acute appendicitis. Among those who underwent laparoscopy, 5.7% had to be converted to open laparotomy. Signs of surgical wound inflammation were found in 10.2% of the patients. Patients who underwent open laparotomy had a significantly higher risk of wound infection (RR=3.1, p-value=0.001). Further analysis revealed an effect modification of pre-operative shaving. Open laparotomy has a higher risk of wound infection among patients who have not had pre-operative shaving (RR=4.1 vs. RR=2.6), while both risks were statistically significant (p-value=0.033 and p-value=0.035), respectively. Complicated cases in intra-operative findings were found to have a higher risk of post-appendectomy SSI.

Conclusion

This study demonstrates that laparoscopic appendectomy carries a lower risk of surgical site infection (SSI) compared to open laparotomy. Additionally, pre-operative shaving of the surgical site was found to increase the incidence of SSI. Healthcare providers can use this information to enhance their practice and reduce the occurrence of surgical site infections. Whenever possible, laparoscopic appendectomy should be preferred over open laparotomy due to its substantially lower SSI risk. We also recommend vigilant monitoring of complicated appendectomy, particularly in cases of ruptured appendicitis, for signs of SSI.

## Introduction

Acute appendicitis is a common surgical emergency. Appendectomy for acute appendicitis is one of the most commonly performed types of emergency surgery, with over 300,000 appendectomies performed annually in the United States [1]. In Riyadh, between 1999 and 2003, 852 patients underwent appendectomy at King Khalid University Hospital [2].

Surgical site infection (SSI) is a common complication of any surgical intervention and can be avoided. SSI is one of the main causes of postoperative morbidity and mortality [3]. According to the Centers for Disease Control and Prevention (CDC), SSI is an infection that occurs after surgery in the part of the body where the surgery took place. SSIs can be superficial infections involving the skin only or more serious infections involving tissues under the skin, organs, or implanted material [4].

Despite advances in infection control practices, including improved operating room ventilation, sterilization methods, barriers, surgical technique, and availability of antimicrobial prophylaxis, SSIs remain a substantial cause of morbidity, prolonged hospitalization, and death. SSI accounts for 20% of all healthcare-associated infections (HAIs) and is associated with a 2- to 11-fold increase in the risk of mortality, with 75% of SSI-associated deaths directly attributable to the SSI [5]. Surgical site infection is the most costly HAI type, with an estimated annual cost of $3.3 billion and extending hospital length of stay by 9.7 days, with the cost of hospitalization increasing by more than $20,000 per admission [5,6]. A systematic review of 35 studies found that the overall rate of infection for open appendectomies was 17.9% (CI: 95% with 10.4-25.3 infections/100 procedures), and the percentage for laparoscopic appendectomy was 8.8% (CI: 95% with 4.5-13.2 infections/100 procedures) [6].

The choice of laparoscopic vs. open method to manage acute appendicitis is usually based on patient-specific factors, such as physical build, cosmetic goals, especially in female patients, old age, and suspected complicated appendix on radiological study peri-operatively. The laparoscopic approach is preferred due to early postoperative recovery and less wound infection, as supported by the literature [7,8].

Despite significant efforts to prevent postoperative complications and increased knowledge regarding preventive measures against surgical site infections (SSI), the burden of this complication remains a major reason for morbidity and longer hospital stays. Identifying groups at risk and predisposing factors can reduce the burden of the disease and lead to clinical recommendations that will reduce the prevalence of SSI. Therefore, the aim of this study is to compare the risk of developing SSI between open laparotomy and laparoscopic appendectomies in Al-Baha, Saudi Arabia.

## Materials and methods

Study setting

This retrospective cohort study compares laparotomy and laparoscopy in terms of postoperative surgical site infection among patients who underwent an appendectomy at King Fahad Hospital (KFH) in Albaha, Saudi Arabia. King Fahad Hospital is a tertiary center that receives patient referrals from eight various provinces that are affiliated with the Albaha region.

Study population

All the patients who underwent appendectomy from January 2018 to December 2022 were eligible for inclusion. Immunocompromised patients "receiving immunosuppression therapy, chemotherapy, or diagnosed with HIV" and those who had appendectomy for an indication other than appendicitis were excluded from the study. The exposure group was defined as patients having an elective laparotomy for appendectomy of both genders, while controls included those who underwent laparoscopy of the same group during the study period.

The sample size was calculated using an online calculator (riskcalc.org). The equation was built with 80% power, 0.05 type I error, unexposed to the exposed ratio of three, and a relative risk of 10. The relative risk was used according to a previous study having a similar objective to the current study [9]. The calculated sample size was about 226. Due to the scarcity of Laparoscopy cases in the selected study period, further inclusion from the years of 2018 and 2019 was done to satisfy the calculated sample size.

Medical record numbers (MRN) of patients who met the inclusion criteria were collected to build the sampling frame. After collecting eligible patients in a sampling frame, simple random sampling using a random number generator was used to select the controls who underwent laparoscopic appendectomy. During the sampling procedure, a considerable exposure to control ratio of 1:3 was used.

To diagnose surgical site infection, the clinical team examined patients using CDC criteria. These criteria include signs and symptoms such as localized pain or tenderness, localized swelling, erythema, heat, and infection involvement or drainage.

Data collection

Data were collected from the surgical health records of the patients. An electronic instrument was designed to store the data of the selected sample. The collected data included patients' demographics, comorbidities, presenting symptoms, ordered imaging studies, pre-operative shaving, type and duration of surgery, intra-operative findings, and signs of wound inflammation. The data collection took place from November 2022 to January 2023.

Statistical analysis

The data analysis was performed using the Statistical Package for the Social Sciences version 29.0 (IBM Inc.. Armonk, New York). Categorical variables were summarized by frequencies and proportions, while continuous variables were summarized by the median and interquartile range (IQR) after applying the Shapiro-Wilk normality distribution test. The exposure variable was the type of surgery (open laparotomy vs. laparoscopy), and the event of interest was the development of signs of surgical wound inflammation. Baseline characteristics and categorical variables were compared using the Chi-squared test. Significant associations with the outcome variable were tested and treated as possible confounders, and significant differences across were taken into consideration and reported. Due to the low number of events, multiple adjustments of confounders were omitted.

Ethical considerations

The scientific research committee affiliated with the education, training center, and academic affairs of King Fahad Hospital in Albaha, Saudi Arabia, granted ethical approval for this study (Ref number KFHIRB/0311202119). After data collection was complete, all personal identifiers were removed and replaced with serial numbers prior to data analysis. The collected information was used solely for research purposes while ensuring patient privacy and confidentiality.

## Results

The total included in the analysis was (256) patients who underwent surgery for acute appendicitis. The age ranged from seven to 91 years with a median and IQR of (29, 21-36). Of the total, 57.4% were males, and 74.6% were Saudis. The most common comorbidity was diabetes mellitus among 4.7%, followed by hypertension at 2.7%. Open surgery was performed on 24.2% of the total, while 75.8% underwent laparoscopy. Of those who underwent laparoscopy, 2.1% were converted to open laparotomy. See the comparison of the baseline characteristics shown in Table 1.

**Table 1:** Comparison of the baseline characteristics between open laparotomy and laparoscopy surgeries of acute appendicitis patients

	Open laparotomy	Laparoscopic	p-value
Gender			
Male	48 (32.7%)	99 (67.3%)	<0.001
Female	14 (12.8%)	95 (87.2%)	
Nationality			
Saudi	44 (23%)	147 (77%)	0.275
Non-Saudi	18 (27.7%)	47 (72.3%)	
Diabetes mellitus			
Yes	5 (41.7%)	7 (58.3%)	0.17
No	57 (23.4%)	187 (76.6%)	
Hypertension			
Yes	3 (42.9%)	4 (57.1%)	0.634
No	59 (23.7%)	190 (76.3%)	
Cardiovascular diseases			
Yes	2 (33.3%)	4 (66.7%)	0.448
No	60 (24%)	190 (76%)	
Coagulopathy			
Yes	3 (50%)	3 (50%)	0.155
No	59 (23.6%)	191 (76.4%)	
Other comorbidities			
Yes	5 (20.8%)	19 (79.2%)	0.806
No	57 (24.6%)	175 (75.4%)	

Approximately half of the patients (49.6%) had symptoms within 24 hours of presentation. The most commonly reported symptom was tenderness in the right lower quadrant, with a frequency of 96.1%. Nausea and vomiting were reported by 55.9% of patients, while anorexia was reported by 48.4% of patients. Migration of pain was reported by 75.8% of patients. Rebound pain was reported by 67.2% of patients. Elevated temperature was the least commonly reported symptom, with a frequency of 11.3%. Computed tomography (CT) was done for two-thirds of the patients (68%), ultrasound was done for 28.1%, X-ray for 28.1%, and magnetic resonance imaging was done for only one patient (0.4%). In CT, fat stranding was the most common finding (46.1%), followed by lymph node enlargement and fluid around the appendix among 27.7% and 23.4%, respectively. See CT findings shown in Table 2.

**Table 2:** Computed tomography findings for patients diagnosed with acute appendicitis

CT findings	N	%
Fat stranding	118	46.10%
Lymph node enlargement	71	27.70%
Fluid around appendix	60	23.40%
Cecal thickness	25	9.80%
Appendix not seen	16	6.30%
Fecalith	14	5.50%
Ileum thickness	14	5.50%
Unremarkable	6	2.30%

**Figure 1:**
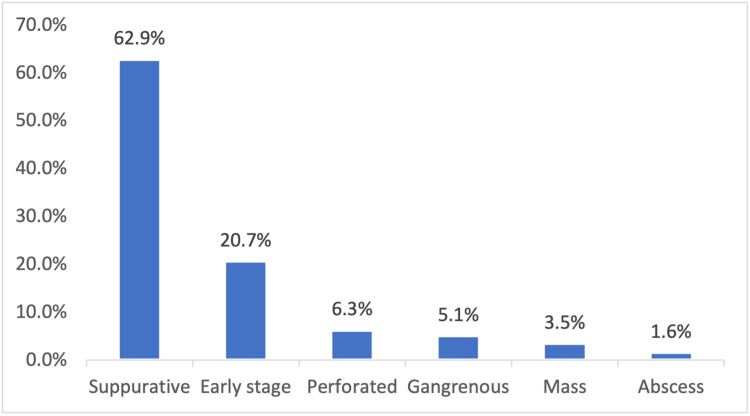
Intraoperative findings of acute appendicitis patients

Pre-operative shaving was done for 34.8% of the patients using razors. Of all the patients who were included in the study, 89.5% received more than one antibiotic. Out of all the surgeries that were carried out, 75.8% were performed using laparoscopic techniques. Additionally, 5.7% of the laparoscopic cases had to be converted to open surgery. See the surgery type and duration in (Table 3). Among all patients included in the study, a majority of surgeries, specifically 69.2%, were completed in under 60 minutes. For 30% of patients, surgeries lasted between 60 and 90 minutes. A small percentage, only 0.8%, of surgeries took longer than 90 minutes to complete. Intra-operative findings revealed that 62.9% of the patients had suppurative appendicitis, 20.7% had early-stage appendicitis, and 16.4% had complicated appendicitis. Complicated cases were those that were perforated, gangrenous, or manifested mass or abscess. On the other hand, suppurative and early-stage cases were considered uncomplicated. For more detailed intra-operative findings, refer to Figure 1.

**Table 3:** Surgery types distribution among patients of acute appendicitis

	N	%
Surgery type		
Open	62	24.20%
Laparoscopic	194	75.80%
Laparoscopic cases converted to open surgery		
Yes	11	5.70%
No	183	94.30%

All included patients had a histopathological diagnosis of appendicitis. Additionally, other histopathological findings were discovered in 31.3% of the patients. The length of stay (LOS) postoperatively was more than 24 hours for the majority of patients (70.7%). Surgical site infections were found among 10.2% of the patients.

Patients who underwent open laparotomy had a significantly higher risk of wound infection (RR=3.1, p-value=0.001). Further analyses revealed an effect modification of pre-operative shaving. Open laparotomy has more risk of wound infection among patients who have not had pre-operative shaving (RR=4.1 vs. RR=2.6), while both risks were statistically significant, p-value=0.033 and p-value=0.035, respectively. The results on the risk of the type of surgery and pre-operative shaving are further detailed in Table 4.

**Table 4:** Total and stratified analysis of the relation between surgery type, pre-operative shaving, and surgical site infection among patients of acute appendicitis

		Surgical site infection		RR (95%CI)
		Yes	No	
Surgery type (Total)	Open laparotomy	13 (21%)	49 (79%)	3.1 (1.5-6.4)
	Laparoscopic surgery	13 (6.7%)	181 (93.3%)	
Stratified analysis				
Pre-operative shaving (Yes)	Open laparotomy	8 (34.8%)	15 (65.2%)	2.6 (1.1-5.83)
	Laparoscopic surgery	9 (13.6%)	57 (86.4%)	
Pre-operative shaving (No)	Open laparotomy	5 (12.8%)	34 (87.2%)	4.1 (1.2-14.5)
	Laparoscopic surgery	4 (3.1%)	124 (96.9%)	

Of the complicated cases, 31% manifested surgical site infections compared to 6.1% of the uncomplicated cases. Therefore, it was found that complicated cases in intra-operative findings had a higher risk of surgical site infection (RR=5.1). Stratified analysis was conducted to show the effect of surgery type on surgical site infection among both groups of intra-operative findings (complicated and uncomplicated), which revealed a small difference between the groups in the RR, 2.6 and 2.3, respectively. The association between laparotomy and surgical site infection stratified by intra-operative findings was significant among the complicated group only. This suggests that complicated appendicitis can be considered an independent risk factor for surgical site infection.

## Discussion

Surgical site infections remain a significant health problem that costs more than 320 million dollars in developed countries [10]. However, the cost can vary greatly depending on the incidence rate from country to country, which can lead to higher costs in lower-income countries. The overall incidence of SSI was estimated to range from zero to 37.4, with a pooled estimate of 7/100 appendectomies, as reported in a meta-analysis [11]. 

Our study, which included 256 patients, indicates that open laparotomy is associated with a higher risk of surgical site infection (SSI) among patients undergoing appendectomy. Those who undergo laparotomy have three times the risk of SSI compared to laparoscopic appendectomy. Similar findings were reported in a local study in Saudi Arabia, which found that SSI had significantly higher odds among laparotomy patients (OR=4.11) [12]. Furthermore, a systematic review found that the pooled SSI incidence was 17.9/100 open laparotomies compared to 8.8/100 laparoscopic appendectomies [7]. On a larger scale, a meta-analysis also reported a discrepancy in SSI rates between open laparotomy and laparoscopic procedures (11 vs. 4.6) per 100 appendectomies, respectively [11]. Although not statistically significant, another study reported that laparotomy had higher rates of wound infection compared to laparoscopy (15.9% vs. 6.8%, respectively) [13]. In contrast to our results, Thomson et al. reported that wound sepsis, procedure duration, and length of hospital stay did not show a statistical difference between open and laparoscopic appendectomies [14]. 

Further analysis of our results revealed that pre-operative shaving of the surgical site contributes to the incidence of surgical site infections (SSIs). Our findings indicate that both laparoscopy procedures and hair removal can significantly reduce the incidence of SSIs. In contrast to our results, previous studies have indicated that pre-operative shaving does not increase the risk of SSIs nor provide a preventive effect against them [15,16]. Although other studies have considered different methods of hair removal, the use of clippers or chemical creams for hair removal had moderate certainty evidence of reducing the incidence of SSIs compared to using a razor for hair removal [17]. 

Complicated appendectomy carries a higher risk of post-appendectomy surgical site infection (SSI) independent of other factors, particularly evident among cases with ruptured appendicitis [18]. A systematic review comparing delayed and primary wound closure for patients with contaminated wounds or complicated appendicitis concluded that there is no superior method to reduce SSI [19]. Our results indicate that the risk difference in SSI between complicated and non-complicated appendicitis was not substantial (2.6% vs. 2.3%). However, the risk of SSI was statistically significant among those with complicated appendicitis, which is consistent with previous studies identifying complicated appendicitis as an independent risk factor for SSI [18,19]. Our findings align with those of Foster et al., who reported higher pooled SSI rates among complicated cases (24.9% vs. 10.5%) [7].

The use of electronic instruments for standardized data collection reduced potential bias and increased accuracy and efficiency. This study examined an important topic and provided valuable information for healthcare providers to improve their practice and reduce surgical site infections. However, the study has limitations. As a single-center study, the findings may not be generalizable to other settings. The retrospective design may have limited data availability and quality. The study did not investigate other potential risk factors for surgical site infections, such as obesity or smoking. Additionally, the study did not consider the cost analysis of different surgical approaches, which could be an important factor for patients and healthcare systems in decision-making.

## Conclusions

The study indicates that laparoscopic appendectomy is associated with a lower risk of surgical site infection (SSI) than open laparotomy. Furthermore, it has been discovered that shaving the surgical site before an operation can increase the likelihood of surgical site infections (SSI). Additionally, complicated appendectomy carries an independent higher risk of post-appendectomy SSI. Healthcare providers can use this information to improve their practice and reduce surgical site infections. Whenever possible, laparoscopic appendectomy is preferred over open laparotomy due to its significantly lower risk of SSI. We also recommend close monitoring of complicated appendectomy, especially in cases of ruptured appendicitis, for signs of SSI. By following these recommendations, healthcare providers can improve their practice and reduce surgical site infections, ultimately leading to better patient outcomes.
